# Prospective Evaluation of the Addition of Polygenic Risk Scores to Breast Cancer Risk Models

**DOI:** 10.1093/jncics/pkab021

**Published:** 2021-03-02

**Authors:** Sherly X Li, Roger L Milne, Tu Nguyen-Dumont, Xiaochuan Wang, Dallas R English, Graham G Giles, Melissa C Southey, Antonis C Antoniou, Andrew Lee, Shuai Li, Ingrid Winship, John L Hopper, Mary Beth Terry, Robert J MacInnis

**Affiliations:** 1 Cancer Epidemiology Division, Cancer Council Victoria, Melbourne, Victoria, Australia; 2 Centre for Epidemiology and Biostatistics, University of Melbourne, Melbourne, Victoria, Australia; 3 Medical Research Council Epidemiology Unit, University of Cambridge, Cambridge, UK; 4 Precision Medicine, School of Clinical Sciences at Monash Health, Monash University, Melbourne, Victoria, Australia; 5 Department of Clinical Pathology, The University of Melbourne, Melbourne, Victoria, Australia; 6 Centre for Cancer Genetic Epidemiology, Department of Public Health and Primary Care, Strangeways Research Laboratory, University of Cambridge, Cambridge, UK; 7 Department of Genomic Medicine, Royal Melbourne Hospital, Melbourne, Victoria, Australia; 8 Department of Medicine, Royal Melbourne Hospital, University of Melbourne, Melbourne, Victoria, Australia; 9 Department of Epidemiology, Mailman School of Public Health, Columbia University, New York, USA

## Abstract

**Background:**

The Breast and Ovarian Analysis of Disease Incidence and Carrier Estimation Algorithm and the International Breast Cancer Intervention Study breast cancer risk models are used to provide advice on screening intervals and chemoprevention. We evaluated the performance of these models, which now incorporate polygenic risk scores (PRSs), using a prospective cohort study.

**Methods:**

We used a case-cohort design, involving women in the Melbourne Collaborative Cohort Study aged 50-75 years when surveyed in 2003-2007, of whom 408 had a first primary breast cancer diagnosed within 10 years (cases), and 2783 were from the subcohort. Ten-year risks were calculated based on lifestyle factors, family history data, and a 313-variant PRS. Discrimination was assessed using a C-statistic compared with 0.50 and calibration using the ratio of expected to observed number of cases (E/O).

**Results:**

When the PRS was added to models with lifestyle factors and family history, the C-statistic (95% confidence interval [CI]) increased from 0.57 (0.54 to 0.60) to 0.62 (0.60 to 0.65) using IBIS and from 0.56 (0.53 to 0.59) to 0.62 (0.59 to 0.64) using BOADICEA. IBIS underpredicted risk (E/O = 0.62, 95% CI = 0.48 to 0.80) for women in the lowest risk category (<1.7%) and overpredicted risk (E/O = 1.40, 95% CI = 1.18 to 1.67) in the highest risk category (≥5%), using the Hosmer-Lemeshow test for calibration in quantiles of risk and a 2-sided *P* value less than*  *.001. BOADICEA underpredicted risk (E/O = 0.82, 95% CI = 0.67 to 0.99) in the second highest risk category (3.4%-5%); the Hosmer-Lemeshow test and a 2-sided *P* value* *was equal to .02.

**Conclusions:**

Although the inclusion of a 313 genetic variant PRS doubles discriminatory accuracy (relative to reference 0.50), models with and without this PRS have relatively modest discrimination and might require recalibration before their clinical and wider use are promoted.

Breast cancer (BC) is the most common cancer and cause of cancer death for women worldwide with approximately 2.1 million incident cases in 2018 (1), a substantial burden of disease (2). Early detection by screening is a key strategy to reduce this burden (3).

Mammographic screening of women aged 50 years and older reduces breast cancer mortality (4). Refining eligibility and employing more tailored screening intervals might lead to earlier cancer detection. Several BC risk models exist (5) that can be used to stratify women and inform risk-tailored advice on the optimal age range, frequency, and modality of screening (6). Even in the absence of detecting a pathogenic variant, these models are used to stratify risk management approaches. There is also considerable value in applying risk models to the general population for targeted screening and chemoprevention.

A recent evaluation of 4 commonly used models, using a sample enriched for having a family history of BC, found that the International Breast Cancer Intervention Study (IBIS) model (7) and the Breast and Ovarian Analysis of Disease Incidence and Carrier Estimation Algorithm (BOADICEA) model (8,9) performed best in terms of calibration and discrimination (10). Given that most women who are screened for BC are older than 50 years, independent prospective studies of how different risk models perform over a longer follow-up period in this age group within an average-risk setting are needed.

Inclusion of common single nucleotide polymorphisms (SNPs) associated with BC into risk models is likely to enhance their performance. More than 160 SNPs have been found to be associated with BC risk at a *P* value less than  5 x 10^-8^ (11), and polygenic risk scores (PRSs) based on these risk-associated markers improve risk stratification (12-15). These PRSs explain a substantial proportion of familial risk (11), more so at an older age, whereas rare moderate- and high-risk germline variants in the major BC susceptibility genes explain a greater proportion of familial risk at a younger age (16).

The latest versions of IBIS and BOADICEA have both incorporated a PRS into their predictions (distinct from additions post hoc), but these updated models have yet to be prospectively evaluated, particularly regarding calibration.

We aimed to evaluate if the addition of PRSs based on SNPs to absolute risk estimates from the IBIS and BOADICEA models adds value to discrimination and calibration using an independent prospective community-based cohort study.

## Methods

### Study Design and Participants

The Melbourne Collaborative Cohort Study (MCCS) is a prospective cohort that includes 24 469 women from Melbourne, Australia, aged between 27 and 76 years (99% were 40-69 years) at recruitment (17). All participants were of White European descent, including 12% born in Italy, 10% in Greece, and 7% in the United Kingdom, and had attended baseline (1990-1994) and up to 2 additional waves of active follow-up (1 in 1995-1998 and/or 1 in 2003-2007). Our analyses included women who were aged 50-75 years when they attended follow-up 2 (2003-2007; designated as the start of follow-up for this analysis), because this age range aligns with current eligibility for government-funded mammographic screening in Australia (18) and follow-up 2 had the most complete data available. Women were eligible if they had completed the baseline and follow-up 2 questionnaires and had no prevalent breast or ovarian cancer prior to their follow-up 2 visit (n = 12 673).

We used a case-cohort design to be more cost efficient than genotyping the whole cohort (19). Supplementary Figure 1 (available online) shows that the case cohort consisted of 3098 women, comprising 408 women diagnosed with a first invasive BC within 10 years after follow-up 2 visit and a random sample of women attending follow-up 2 (hereafter called the subcohort) of 2783 women (22% of the whole female cohort) of whom 93 were cases. Simulations had shown that this was an optimal cost-effective sampling fraction to minimize the parameter variances of interest (20). MCCS participants provided informed consent, and the Cancer Council Victoria Human Research Ethics Committee approved the study (17).

### Risk Assessment

We used the latest versions (at the time of analysis) of the risk models: BOADICEA version 5.0.0 (8,9) and IBIS version 8 b (7). These models varied in their prediction period, underlying age-specific incidences of BC, and predictors (Supplementary Table 1, available online).

At follow-up 2, MCCS participants completed a lifestyle questionnaire that asked about their demographic characteristics including age, alcohol intake, age at menarche, parity, number of sisters and children, age at first birth, menopausal status, and use of oral contraceptive pill and menopausal hormone therapy. Summary family history data on affected relatives were obtained from questionnaires at follow-up 2 (first-degree relatives) and follow-up 1 (aunts and grandmothers). Data from the most recent questionnaires were used and supplemented with that from older questionnaires if unavailable. To reconstruct pedigrees, the following assumptions were made about the year of birth (YOB) of participants’ relatives: mothers and aunts (25 years before the participant’s YOB), grandmothers (50 years before the participant’s YOB), sisters (participant’s YOB), and daughters (25 years after the participant’s YOB). Missing ages for affected and unaffected mothers, aunts, and grandmothers were imputed to 70 years, whereas sisters were imputed to the youngest of participant age at follow-up 2 or age 70 years. Weight at follow-up 2 was measured to the nearest 100 g using a digital electronic scale, and height was measured at baseline to the nearest 1 mm, using a stadiometer. Body mass index was defined as weight (kg) divided by height squared (m^2^).

Mammography density measures, results from germline genetic testing for *BRCA1* and *BRCA2* (or other rare variants), and history of hyperplasia were unavailable for most female participants in the MCCS so were not included in our analyses.

### Polygenic Risk Score

We genotyped all 3098 case-cohort participants using the Illumina Infinium OncoArray-500K BeadChip and imputed the missing autosome SNPs using the Michigan imputation server with the 1000 Genomes Project reference panel (phase 3) (21). We included SNPs that had genotype call rates of 95% or more, imputation *R*^2^ of 0.3 or more, and minor allele frequency of 0.1% or more. Post-quality control SNPs were used to generate a PRS based on the genome-wide association study results published by the Breast Cancer Association Consortium (BCAC) (11,13). The same set of 313 SNPs and per allele odds ratio (using BCAC estimates) were used for both IBIS and BOADICEA PRS (13); however, model-specific methods were used to construct them. The PRS in the BOADICEA model was calculated by summing across variants the product of the per allele log-odds ratio and the effective allele counts for each SNP (using BCAC estimates) and then normalized using a population-based underlying risk and allele frequency (9). The PRS for IBIS was calculated using the relative risk of developing BC for each genotype, estimating the average population relative risk accounting for the population-based risk and allele frequency, applying this to the women’s genotype, and then multiplying the SNP-specific relative risks together (22).

### Outcome Assessment

Incident cases and vital status were ascertained from record linkage between the Victorian Cancer Registry; the Victorian Registry of Births, Deaths and Marriages; the National Death Index; and the Australian Cancer Database. Cases were women notified to the registry with a first diagnosis of invasive adenocarcinoma of the breast (Third Revision of the International Classification of Diseases for Oncology code C50) during follow-up to June 30, 2016.

### Statistical Analysis

Follow-up began from age at follow-up 2 attendance and ended at 1) diagnosis of invasive BC, 2) follow-up time reaching 10 years, 3) age 80 years (maximum age for estimating risk in BOADICEA), or 4) censor date of June 30, 2016, whichever came first. Expected risk for the subcohort was estimated by summing the percentage risk from outputs of IBIS or BOADICEA for participants in the subcohort and then dividing by the sampling fraction (0.22) used to select the subcohort. Death from causes other than BC was a competing risk, with no censoring applied at death from other causes in the main analysis.

We compared the performance of the models up to 10-year risk in terms of discrimination and calibration. Calibration was assessed by comparing the number of expected cases (E) within the whole cohort with the number observed (O), where E was calculated as the number expected in the subcohort multiplied by the inverse of the sampling fraction. We calculated a robust 95% confidence interval (CI) for E/O by:

$$\frac{E}{O}\pm \sqrt{(\mathrm{Var}(\log\left(\frac{E}{O})\right)}.$$

Where $\mathrm{Var}\left(\log\left(\frac{E}{O}\right)\right)=\frac{1}{O}+\frac{\mathrm{Var}(\overset{-}{E})}{{\overset{-}{E}}^{2}},\;\overset{-}{E}$ is the mean expected cases in the subcohort, and Var($\overset{-}{E}$) is the finite sample variance of the mean expected cases from the subcohort.

Model discrimination was assessed using a concordance statistic (C-statistic) (23) and plotting the receiver operating characteristic curve, accounting for incomplete follow-up, where 1 indicates perfect discrimination and 0.50 indicates discrimination no better than chance. We compared models (accounting for correlation between models) using the Wald test with inclusion of the following components: family history, lifestyle factors, and PRS sequentially.

Model calibration and discrimination were also examined by categories of model-specific 10-year risk (quantiles), stratified by age (50-64 and 65 years and older, because women in the latter group can be eligible for universal health care in some countries) (24) and by whether the women had an affected first- or second-degree relative. We also examined model performance for a shorter period of risk (5 years). Sensitivity analyses included censoring at diagnosis of ductal carcinoma in situ during follow-up (6 cases), accounting for competing risk of death because of other causes (for IBIS; BOADICEA does not provide this option), and by applying updated Australian BC population incidence rates for BOADICEA that take into account changes between 2010 and 2015 (25). The heterogeneity of calibration across quantiles of risk was assessed using the Hosmer-Lemeshow test.

We calculated the specificity of all risk models at fixed sensitivity levels based on the full model (family history, lifestyle factors, and PRS) at a threshold of 3.4% for 10-year BC risk (26,27). The 3.4% threshold corresponds to the 10-year risk of an average 60-year-old woman and is approximately double the 5-year risk of 1.67% that has been used to define high risk for the purpose of eligibility in some chemoprevention trials. We calculated the mean risk stratification (MRS), comparing models with and without PRS (28). Analyses were performed using Stata (version 16) and R (version 3.5.1).

## Results

Characteristics of study participants in the case cohort are shown in Table 1. Women in the random subcohort were representative of those from the full cohort, with similar incidence of BC. The 10-year risk with all predictors (including PRS) had wider ranges than the models with family history and lifestyle factors alone (Supplementary Figure 2, available online).

**Table 1.** Melbourne Collaborative Cohort Study participant characteristics

Characteristics	Cases of BC (n = 408)	Subcohort (n = 2783)	Whole cohort (n= 12 673)
Mean age (SD), y	63.0 (6.9)	63.6 (7.2)	63.5 (7.2)
Mean height (SD), cm	161.8 (6.2)	161.1 (6.5)	161.0 (6.6)
Mean weight (SD), kg	73.0 (13.3)	70.6 (13.5)	70.8 (13.6)
Mean BMI (SD), kg/m^2^	27.9 (5.3)	27.2 (5.2)	27.4 (5.3)
Mean alcohol intake (SD), ethanol g/day	8.6 (11.1)	8.1 (11.4)	8.0 (11.5)
Mean menarche age (SD), y	12.8 (1.5)	13.0 (1.6)	13.0 (1.6)
Mean No. of live births (SD)	2.3 (1.5)	2.4 (1.5)	2.4 (1.5)
Mean age at first birth (SD), y	25.5 (4.7)	25.2 (4.5)	25.2 (4.6)
Mean age of menopause (SD), y^a^	50.1 (5.1)	49.7 (5.0)	49.6 (5.0)
Mean incidence of breast cancer^b^ per 1000 person-years (95% CI)	—	1.17 (0.96 to 1.43)	1.13 (1.02 to 1.24)
Oral contraceptive use, No. (%)			
Never	110 (27.0)	830 (29.8)	3766 (29.7)
Former	296 (72.5)	1937 (69.6)	8858 (69.9)
Current	2 (0.5)	12 (0.4)	37 (0.3)
Missing	—	4 (0.1)	12 (0.1)
Menopausal status,^c^ No. (%)			
Premenopausal	344 (84.3)	7 (0.3)	37 (0.3)
Postmenopausal	64 (15.7)	2425 (87.1)	11 025 (87.0)
Missing	—	1 (0.0)	3 (0.0)
Unable to determine	—	350 (12.6)	1608 (12.7)
Menopausal hormone therapy use,^d^ No. (%)			
Never	184 (45.1)	1424 (51.2)	6524 (51.5)
Former	200 (49.0)	695 (25.0)	3101 (24.5)
Current estrogen	8 (2.0)	32 (1.1)	151 (1.2)
Current other hormone replacement therapy	61 (15.0)	222 (8.0)	939 (7.4)
Current user but missing type	20 (4.9)	144 (5.2)	680 (5.4)
Missing	35 (8.6)	266 (9.6)	1278 (10.1)
Family history of breast cancer (first or second degree), No. (%)			
No	292 (71.6)	2185 (78.5)	9925 (78.3)
Yes	116 (28.4)	598 (21.5)	2748 (21.7)
Mean PRS distribution (SD)			
IBIS	0.12 (0.64)	−0.12 (0.62)	—
BOADICEA	0.50 (1.05)	0.09 (1.02)	—

a Women whose reason for periods stopping were due to having had a natural menopause or a bilateral oophorectomy. — = not applicable; BC = breast cancer; BOADICEA = Breast and Ovarian Analysis of Disease Incidence and Carrier Estimation Algorithm model (version 5.0.0); IBIS = International Breast Cancer Intervention Study model (version 8 b); PRS = polygenic risk score based on 313 single nucleotide polymorphisms associated with breast cancer.

b Standardized incidence rate.

c Postmenopausal is defined as had menstrual period in last 12 months and currently using hormone replacement therapy (or missing) and aged at least 55 years; or no menstrual period in last 12 months (or missing) and periods stopped naturally; or no menstrual period in last 12 months (or missing) and periods stopped because both ovaries were removed; or no menstrual period in last 12 months (or missing) and periods stopped because of hysterectomy or other reason (or missing) and aged at least 55 years.

d Type of hormone replacement therapy based on assumption of estrogen for those who have had a hysterectomy and combined estrogen and progesterone for those on hormone replacement therapy but have not had a hysterectomy.

**Figure 1.**
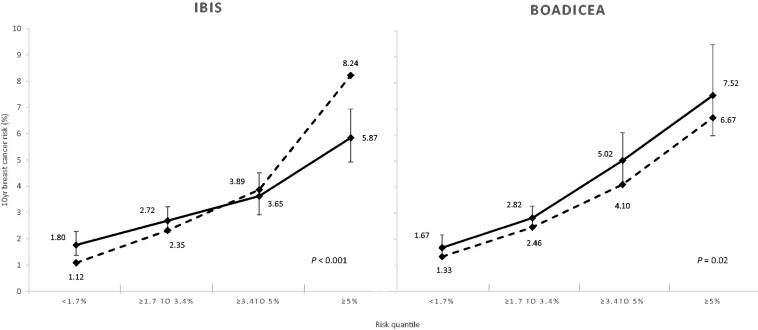
Calibration of 10-year breast cancer risk scores for IBIS and BOADICEA models by quantile of risk. The **dashed line** represents the predicted risk. The **solid line** represents the observed cumulative incidence. The models include age, family history, lifestyle factors, and polygenic risk score, based on the case cohort (n = 3098). For more detailed estimates, see Supplementary Table 2 (available online). Categorization is based on the distribution of raw 10-year breast cancer risk for each of the respective risk prediction models. Numbers and estimates are based on up to 10-year breast cancer risk, which has been adjusted for length of follow-up. Two-sided *P* values represent the Hosmer-Lemeshow test statistic across all 4 risk quantiles. BOADICEA = Breast and Ovarian Analysis of Disease Incidence and Carrier Estimation Algorithm model (version 5.0.0); IBIS = International Breast Cancer Intervention Study model (version 8 b).

Overall, for models using all predictors for which data were available (age, family history, lifestyle factors, and PRS), the E/O for BOADICEA was 0.85 (95% CI = 0.77 to 0.94), whereas for IBIS, it was 1.06 (95% CI = 0.95 to 1.17) (Table 2). IBIS underpredicted risk (E/O = 0.62, 95% CI = 0.48 to 0.80) for women in the lowest risk category (<1.7%) and overpredicted risk (E/O = 1.40, 95% CI = 1.18 to 1.67) in the highest risk category (≥5%); Hosmer-Lemeshow test for calibration in quantiles of risk, 2-sided *P* value <.001. BOADICEA underpredicted risk (E/O = 0.82, 95% CI = 0.67 to 0.99) in the second highest risk category (3.4%-5%); Hosmer-Lemeshow test, 2-sided *P *value equal to * *.02 (Figure 1; Supplementary Table 2, available online).

**Table 2.** Calibration and discrimination statistics for IBIS and BOADICEA 10-year risk scores^a^

Risk model	Case-cohort, No.	Subcohort, No.	Expected No. of cases	Observed No. of cases	Expected/Observed ratio (robust 95% CI)	Concordance statistic (95% CI)
Overall						
IBIS	3098	2783	431.3	408	1.06 (0.95 to 1.17)	0.62 (0.60 to 0.65)
BOADICEA	3098	2783	346.9	408	0.85 (0.77 to 0.94)	0.62 (0.59 to 0.64)
Age 50-64 years at baseline						
IBIS	1732	1549	256.8	235	1.09 (0.95 to 1.25)	0.64 (0.60 to 0.67)
BOADICEA	1732	1549	220.7	235	0.94 (0.82 to 1.07)	0.65 (0.62 to 0.68)
Age 65-75 years at baseline						
IBIS	1366	1234	174.6	173	1.01 (0.86 to 1.18)	0.60 (0.55 to 0.65)
BOADICEA	1366	1234	126.6	173	0.73 (0.63 to 0.85)	0.58 (0.53 to 0.62)
No family history of breast cancer						
IBIS	2401	2185	272.3	292	0.93 (0.83 to 1.05)	0.61 (0.58 to 0.65)
BOADICEA	2401	2185	249.1	292	0.85 (0.76 to 0.96)	0.61 (0.57 to 0.64)
Family history of breast cancer						
IBIS	697	598	159.7	116	1.38 (1.13 to 1.67)	0.63 (0.58 to 0.68)
BOADICEA	697	598	98.1	116	0.85 (0.70 to 1.02)	0.61 (0.56 to 0.66)

a Model: age, family history, lifestyle factors and PRS. CI = confidence interval; BOADICEA = Breast and Ovarian Analysis of Disease Incidence and Carrier Estimation Algorithm model (version 5.0.0); IBIS = International Breast Cancer Intervention Study model (version 8 b); PRS = polygenic risk score based on 313 single nucleotide polymorphisms associated with breast cancer.

**Figure 2.**
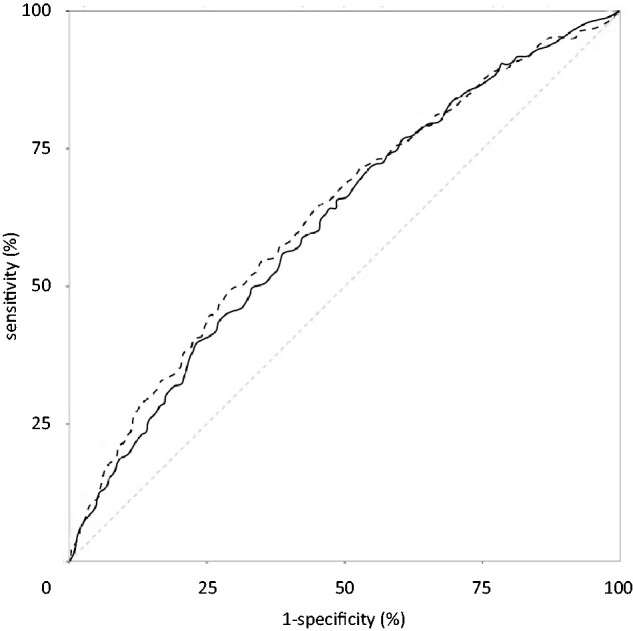
Receiver operating characteristic curves for IBIS (**dashed line**) and BOADICEA (**solid line**) breast cancer risk models (family history, lifestyle factors, and polygenic risk score). The case cohort consisted of 3098 women. The area under the curve was 0.62 (95% confidence interval = 0.60 to 0.65) for IBIS and 0.62 (95% confidence interval = 0.59 to 0.64) for BOADICEA. The **dotted line** represents the line of no discrimination. For more detailed comparisons, see Table 3. BOADICEA = Breast and Ovarian Analysis of Disease Incidence and Carrier Estimation Algorithm model (version 5.0.0); IBIS = International Breast Cancer Intervention Study model (version 8 b).

In terms of discrimination, the C-statistics for the 2 models were similar (Figure 2). For both IBIS and BOADICEA, the addition of a PRS provided double the discriminatory accuracy (from reference 0.50) compared with the model that included family history and lifestyle factors; C-statistics increased from 0.57 (95% CI = 0.54 to 0.60) to 0.62 (95% CI = 0.60 to 0.65) using IBIS, and from 0.56 (95% CI = 0.53 to 0.59) to 0.62 (95% CI = 0.59 to 0.64) using BOADICEA, (*P*_diff_ < .001) (Table 3). The addition of family history made little difference to the model discriminatory ability when compared with models that included age, PRS, and lifestyle factors (*P*_diff_ = .56 for IBIS, *P*_diff_ = .39 for BOADICEA).

**Table 3.** Discrimination statistics for IBIS and BOADICEA 10-year risk scores, by risk models

Variables inputted into the models	IBIS		BOADICEA	
	Concordance statistic (95% CI)	*P* ^a^	Concordance statistic (95% CI)	*P* ^a^
Age	0.50 (0.47 to 0.53)	<.001	0.51 (0.48 to 0.54)	<.001
Age, PRS	0.61 (0.58 to 0.64)	.03	0.59 (0.57 to 0.62)	.02
Age, family history	0.53 (0.50 to 0.56)	<.001	0.52 (0.49 to 0.55)	<.001
Age, lifestyle	0.56 (0.53 to 0.59)	<.001	0.56 (0.53 to 0.59)	<.001
Age, family history, PRS	0.61 (0.58 to 0.64)	.01	0.60 (0.57 to 0.63)	.04
Age, family history, lifestyle	0.57 (0.54 to 0.60)	<.001	0.56 (0.53 to 0.59)	<.001
Age, lifestyle, PRS	0.62 (0.59 to 0.65)	.56	0.61 (0.59 to 0.64)	.39
Age, family history, lifestyle, PRS	0.62 (0.60 to 0.65)	—	0.62 (0.59 to 0.64)	—

a Two-sided *P* values for the Wald test comparing model with all variables included. CI = confidence interval; BOADICEA = Breast and Ovarian Analysis of Disease Incidence and Carrier Estimation Algorithm model (version 5.0.0); IBIS = International Breast Cancer Intervention Study model (version 8 b); PRS = polygenic risk score based on 313 single nucleotide polymorphisms associated with breast cancer.

Table 2 provides an overview of stratified calibration results. IBIS was well calibrated in both age groups, but BOADICEA underpredicted risk for women aged 65 years and older. For women with a family history, the IBIS model underpredicted risk when including only lifestyle factors and PRS (E/O = 0.80, 95% CI = 0.66 to 0.97) but overpredicted risk (E/O = 1.38, 95% CI = 1.13 to 1.67) when BC family history information was included (Supplementary Table 3, available online). This pattern was not observed for BOADICEA.

Findings for IBIS did not differ when we considered different assumptions regarding competing mortality events and after censoring at diagnosis of ductal carcinoma in situ (results not shown). Using updated Australian BC incidence rates reduced the underprediction of overall risk for BOADICEA (from E/O = 0.85, 95% CI = 0.77 to 0.94, to E/O = 0.89, 95% CI = 0.80 to 0.98), and E/O did not differ from 1 in any category of predicted risk or when stratified by BC family history (Supplementary Table 4, available online). Results for 5-year risk of BC were in the same direction but had wider confidence intervals (Supplementary Table 5, available online).

When we set a fixed sensitivity equivalent to a 3.4% 10-year risk using full models of IBIS (sensitivity = 55.2%) and BOADICEA (sensitivity = 43.4%), we found that specificity was at least 6.6% higher for IBIS and 10.1% higher for BOADICEA for those models that included PRS compared with their equivalent model that did not include PRS (Table 4). The MRS based on 10-year risk that included PRS varied on average by 1.5% for both models, whereas the MRS without PRS was 0.9% and 0.7% for IBIS and BOADICEA, respectively. The population average 10-year risk for breast cancer was 3.2%.

**Table 4.** Case-cohort sensitivity and specificity for IBIS and BOADICEA 10-year risk scores^a^

Risk model	Threshold for 10-year risk, %	No. of breast cancer cases above the respective threshold	Sensitivity (95% CI), %	Specificity (95% CI), %	Specificity *P*_diff_ compared with family history + lifestyle + PRS model
IBIS					
Family history + lifestyle + PRS	≥3.4	225^b^	55.2 (50.5 to 59.8)	62.8 (60.9 to 64.6)	—
Lifestyle + PRS	≥3.3	225	55.2 (50.5 to 59.8)	64.5 (62.7 to 66.3)	.02
Family history + lifestyle	≥3.0	225	55.2 (50.5 to 59.8)	56.2 (54.3 to 58.1)	<.001
Family history + PRS	≥3.1	225	55.2 (50.5 to 59.8)	62.1 (60.2 to 63.9)	.28
Lifestyle	≥3.1	225	55.2 (50.5 to 59.8)	57.9 (56.0 to 59.8)	<.001
Family history	≥2.6	225	55.2 (50.5 to 59.8)	49.7 (47.8 to 51.6)	<.001
PRS	≥3.0	225	55.2 (50.5 to 59.8)	63.3 (61.5 to 65.2)	.52
BOADICEA					
Family history + lifestyle + PRS	≥3.4	177^b^	43.4 (38.7 to 48.0)	71.9 (70.1 to 73.6)	—
Lifestyle + PRS	≥3.4	177	43.4 (38.7 to 48.0)	71.7 (70.0 to 73.5)	.82
Family history + lifestyle	≥2.9	177	43.4 (38.7 to 48.0)	61.5 (59.6 to 63.3)	<.001
Family history + PRS	≥3.6	177	43.4 (38.7 to 48.0)	67.7 (65.9 to 69.5)	<.001
Lifestyle	≥2.9	177	43.4 (38.7 to 48.0)	61.6 (59.7 to 63.5)	<.001
Family history	≥3.0	177	43.4 (38.7 to 48.0)	52.5 (50.6 to 54.5)	<.001
PRS	≥3.7	177	43.4 (38.7 to 48.0)	69.9 (68.2 to 71.7)	.05

a Case-cohort participants who had genetic data and 10 years of follow up (n = 2580 in the subcohort, 408 breast cancer cases). CI = confidence interval; BOADICEA = Breast and Ovarian Analysis of Disease Incidence and Carrier Estimation Algorithm model (version 5.0.0); IBIS = International Breast Cancer Intervention Study model (version 8 b); PRS = polygenic risk score based on 313 single nucleotide polymorphisms associated with breast cancer.

b Models based on fixed sensitivity for a 10-year risk of breast cancer threshold of ≥3.4% (based on model with all predictors: family history, lifestyle factors, and PRS).

## Discussion

We used prospective data to examine the performances of the current IBIS and BOADICEA models, which now include a PRS based on common genetic variants, to evaluate their potential to inform eligibility for tailored screening and chemoprevention. Using a prospective cohort of women aged 50 years and older, we found that the addition of PRSs improved risk discrimination and that family history offered little additional discriminatory ability to 10-year risk estimates. Overall discrimination, however, was relatively modest. Both models with all predictors were not well calibrated when stratified by risk quantiles according to the Hosmer-Lemeshow test. IBIS underpredicted risk for women in the low-risk categories (<1.7%) and overpredicted risk in the high-risk categories (≥5%). On the other hand, BOADICEA underpredicted risk for women in the second highest category of predicted risk (3.4%-5%). BOADICEA’s calibration improved with updated incidence data.

One reason for differences in calibration between IBIS and BOADICEA could be related to differing PRS implementation. BOADICEA accounts for the contribution of the PRS to the BC familial risk by splitting the polygenic component (capturing unobserved familial effects not due to high- or moderate-risk mutations) into a known component based on the PRS and a residual familial aggregation component (9), thus avoiding double-counting the effect of the SNPs. IBIS, by contrast, treats the observed PRS as an independent risk factor to family history, with no adjustment to the family history component, despite the fact that this PRS explains 18% of the BC familial risk (11). This could explain the overprediction observed with IBIS for women with a family history of BC or for women in the top category of predicted risks.

For models that included PRS and lifestyle factors, the addition of family history contributed little to discrimination. Models with PRS also had higher specificities for a given sensitivity compared with models without PRS, suggesting that adding PRS helps minimize false-positives and reduce overscreening. However, our results show that ignoring family history information can result in substantial underprediction of risk for women with a BC family history. Future research is needed to explore the extent to which the collection of genomic information negates the need to collect family history data for BC risk discrimination and how this depends on the extent of family history data. Genomic data do not change over time so only have to be collected once and are more reliably measured than family history, especially for more distant relatives.

The IBIS model with all predictors overpredicted risk for women with a family history of BC, whereas BOADICEA was reasonably well calibrated. Contrastingly, analyses of the Prospective Family Study Cohort multigenerational family history data found better calibration for women with a family history for IBIS and BOADICEA without the PRS (10). This difference may be because of more complete, extensive, and verified BC family history collection by the Prospective Family Study Cohort, whereas the MCCS relied solely on self-report from participants about BC-affected family members. The MCCS might better reflect how such data are collected in the general population.

This study shows that BC risk models benefit from having the flexibility to update their underlying population-based incidence rates if BC incidence changes over time, as shown from considering calibration using the BOADICEA model. This flexibility enables users to better apply the models to their populations. The importance of using population-specific incidence has been shown previously for the Breast Cancer Risk Assessment Tool (BCRAT) (29,30).

Although we found that discrimination was superior for BC models that included a PRS, cost-benefit analyses are warranted to determine whether such improvements outweigh the burdens on women and clinicians that arise from obtaining genomic information. These burdens include any adverse consequences to a woman’s psychological well-being as well as how genomic information might affect their relatives’ risks of BC. There are currently poor genomic literacy (31) and a lack of benefit in genomic risk disclosure on screening (32) and risk reduction behaviors (33,34). Moreover, the optimal starting age, frequency, and modality of screening will be important factors in determining the utility of PRS-based risk stratification. We look forward to findings from clinical trials examining risk-stratified screening in primary care (6).

Strengths of our study include having PRS and 10 years of prospective follow-up data. Limitations include the lack of complete data for all model inputs, particularly mammographic density and mutation status in high-risk genes (such as *BRCA1* and *BRCA2*). The inclusion of these factors might dilute the effect of adding PRS to the models, depending on family history, although the estimated population frequency of mutation carriers is low (35). Our current evaluation was conducted using a sample of European ancestry, and replication is required for other ethnic groups. Also, these results may not be applicable for women younger than 50 years, who may be candidates for chemoprevention. Evaluation in other populations is warranted. Nevertheless, our examination of this in an average-risk sample helps determine if the models have wider reach beyond high-risk populations.

In conclusion, for Australian women aged 50-75 years, the addition of a 313-variant PRS to current risk models (age, lifestyle, and family history) improves discrimination for estimating 10-year BC risk by twofold (from reference 0.50), although the discrimination remains relatively modest. Family history data do not appear to appreciably improve discrimination once a PRS is included. Both models might need recalibration.

## Funding

This work was primarily supported by grant 1129136 from the Australian National Health and Medical Research Council (NHMRC) (https://www.nhmrc.gov.au/).

MCCS cohort recruitment was funded by Cancer Council Victoria (https://www.cancervic.org.au/) and VicHealth (https://www.vichealth.vic.gov.au/). The MCCS was further supported by Australian NHMRC grants 209057, 396414, and 1074383, and ongoing follow-up and data management have been funded by Cancer Council Victoria since 1995. Cases and their vital status were ascertained through the Victorian Cancer Registry and the Australian Institute of Health and Welfare, including the National Death Index and the Australian Cancer Database.

TN-D is a recipient of a Career Development Fellowship from the National Breast Cancer Foundation (Australia). JLH and MCS are Senior Principal and Senior Research Fellows of the National Health and Medical Research Council (Australia), respectively. SL is a Victorian Cancer Agency Early Career Research Fellow (Australia). AL and ACA are supported by Cancer Research - UK grant C12292/A20861

## Notes

**Role of the funders**: The sponsors had no role in the design and conduct of the study; collection, management, analysis, and interpretation of the data; preparation, review, or approval of the manuscript; and decision to submit the manuscript for publication.

**Disclosures**: The BOADICEA model has been licensed to Cambridge Enterprise for commercialization, with the authors ACA and AL listed as its inventors. These authors may receive royalties in the future if commercialization is realized. All authors declared no conflicts of interest during the conduct of this story outside the grant funding listed in the Funding section.

**Author contributions:** Conceptualization: RJM, IW, MBT, RLM, TN-D; Data curation: SXL, XW; Formal analysis: SXL, RJM; Funding acquisition: RJM, IW, MBT, RLM, TN-D; Methodology: RJM, SXL, ACA, JLH, MBT, SL; Project administration: RJM, RLM, MCS; Resources: RJM, RLM, TN-D, DRE, GGG, MCS, JLH; Software: ACA, AL; Supervision: RJM, RLM, MCS, GGG, JLH, MBT; Writing—original draft: SXL, RJM; Writing—review & editing: all authors. All authors made the decision to submit the manuscript for publication. RJM had full access to all the data in the study and takes responsibility for the integrity of the data and the accuracy of the data analysis.

**Acknowledgements:** We thank Dr Adam Brentnall for comments that greatly improved earlier versions of the manuscript. We also thank the original MCCS investigators and the diligent team, who recruited the participants and who continue working on follow-up, for their contribution. We express our gratitude to the many thousands of Melbourne residents who continue to participate in the study.

## Data Availability

The MCCS data can be made available on request to pedigree@cancervic.org.au.

## Supplementary Material

pkab021_Supplementary_Data
