# Supplementary material for: Prospective Evaluation of the Addition of Polygenic Risk Scores to Breast Cancer Risk Models
Source: JNCI Cancer Spectr. 2021 Mar 2;5(3):pkab021. doi: 10.1093/jncics/pkab021 (PMC8099999; doi:10.1093/jncics/pkab021)
Supplement: pkab021_Supplementary_Data [file pkab021_supplementary_data.pdf]

Supplementary Materials

Title: Prospective evaluation of the addition of polygenic risk scores to breast cancer risk models

Authors: Sherly X Li, Roger L Milne, Tu Nguyen-Dumont, Xiaochuan Wang, Dallas R English, Graham G Giles, Melissa C Southey, Antonis C Antoniou, Andrew Lee, Shuai Li, Ingrid Winship, John L Hopper, Mary Beth Terry, Robert J MacInnis.

This supplementary material has been provided by the authors to give readers additional information about their work.

Supplementary Table 1: Summary of inputs for IBIS and BOADICEA risk models and their availability within the Melbourne Collaborative Cohort Study[a]

Components	IBIS	BOADICEA	Availability in MCCS
Personal information			
Age	●	●	●
Year of birth		●	●
Ashkenazi Jewish heritage	●	●	
Height	●	●	●
Body mass index	●	●	●
Identical twin status		●	
Alcohol intake		●	●
Age at menarche	●	●	●
No. of live births	●	●	●
Age at first birth	●	●	●
Menopausal status	●	●	●
Age at menopause		●	●
Oral contraceptive pill use		●	●
Menopausal hormone therapy use	●	●	●
History of hyperplasia (atypical or typical)	●		
History of lobular carcinoma in situ	●		●
Previous breast biopsy	●		
History of ovarian cancer	●	●	●
Family history			
Breast cancer	●	●	●
Contralateral breast cancer		●	
Ovarian cancer	●	●	
Pancreatic cancer		●	
Prostate cancer		●	●
Age at cancer diagnosis	●	●	●
Mammogram			
Mammographic density	●	●	*

Genetic information			
BRCA1 mutation status	●	●	*
BRCA2 mutation status	●	●	*
PALB2 mutation status		●	
ATM mutation status		●	
CHEK2 mutation status		●	
Polygenic risk score	●	●	●
Follow-up			
Underlying incidence rates	UK 2005-2009 Thames Cancer Registry	Australia: 1982-2010 AIHW	

[a] IBIS: International Breast Cancer Intervention Study model (version 8b), BOADICEA: the Breast and Ovarian Analysis of Disease Incidence and Carrier Estimation Algorithm model (version 5.0.0), MCCS: Melbourne Collaborative Cohort Study, AIHW: Australian Institute of Health and Welfare. * = Not available for most participants eligible for this study so not included in our analyses

Supplementary Table 2: Calibration statistics for IBIS and BOADICEA 10-year risk scores stratified by quantiles of breast cancer risk[a]

Risk model	Case-cohort, No.	Sub-cohort, No.	Expected No. of cases	Observed No. of cases	Expected /Observed ratio (robust 95%CI)
IBIS					
Family history and lifestyle factors					
<1.7%	80	69	5.0	12	0.42 (0.24,0.74)
≥1.7 to 3.4%	2026	1855	204.8	226	0.91 (0.80,1.03)
≥3.4to 5%	615	546	96.6	94	1.03 (0.84,1.26)
≥5%	377	313	95.7	76	1.26 (1.00,1.59)
Family history, lifestyle factors and PRS					
<1.7%	799	748	38.0	61	0.62 (0.48,0.80)
≥1.7 to 3.4%	1079	988	105.4	122	0.86 (0.72,1.03)
≥3.4to 5%	558	502	88.5	83	1.07 (0.86,1.32)
≥5%	662	545	199.4	142	1.40 (1.18,1.67)
BOADICEA					
Family history and lifestyle factors					
<1.7%	518	481	30.5	47	0.65 (0.49,0.86)
≥1.7 to 3.4%	1970	1785	200.4	238	0.84 (0.74,0.96)
≥3.4to 5%	481	413	73.2	91	0.80 (0.65,0.99)
≥5%	129	104	30.5	32	0.95 (0.67,1.35)
Family history, lifestyle factors and PRS					
<1.7%	811	762	46.2	58	0.80 (0.62,1.03)
≥1.7 to 3.4%	1482	1351	151.1	173	0.87 (0.75,1.01)
≥3.4to 5%	527	451	84.1	103	0.82 (0.67,0.99)
≥5%	278	219	65.6	74	0.89 (0.70,1.12)

[a] CI: confidence interval, PRS: polygenic risk score based on 313 single-nucleotide polymorphisms associated with breast cancer, IBIS: International Breast Cancer Intervention Study model (version 8b), BOADICEA: Breast and Ovarian Analysis of Disease Incidence and Carrier Estimation Algorithm model (version 5.0.0).

Supplementary Table 3: Calibration statistics for IBIS and BOADICEA 10-year risk scores stratified by family history of breast cancer[a]

Risk model	Case-cohort, No.	Sub-cohort, No.	Expected No. of cases	Observed No. of cases	Expected /Observed ratio (robust 95%CI)	Concordance statistic (95%CI)
IBIS						
Family history and lifestyle factors						
No	2401	2185	259.6	292	0.89 (0.79,1.00)	0.55 (0.51,0.58)
Yes	697	598	142.0	116	1.22 (1.02,1.47)	0.56 (0.51,0.61)
Lifestyle factors and PRS						
No	2401	2185	303.5	292	1.04 (0.92,1.17)	0.61 (0.58,0.65)
Yes	697	598	92.8	116	0.80 (0.66,0.97)	0.63 (0.58,0.68)
Family history, lifestyle factors and PRS						
No	2401	2185	272.3	292	0.93 (0.83,1.05)	0.61 (0.58,0.65)
Yes	697	598	159.7	116	1.38 (1.13,1.67)	0.63 (0.58,0.68)
BOADICEA						
Family history and lifestyle factors						
No	2401	2185	239.1	292	0.82 (0.73,0.92)	0.55 (0.51,0.58)
Yes	697	598	95.9	116	0.83 (0.69,0.99)	0.56 (0.51,0.62)
Lifestyle factors and PRS						
No	2401	2185	267.4	292	0.92 (0.81,1.03)	0.61 (0.57,0.64)
Yes	697	598	80.3	116	0.69 (0.57,0.83)	0.62 (0.57,0.67)
Family history, lifestyle factors and PRS						
No	2401	2185	249.1	292	0.85 (0.76,0.96)	0.61 (0.57,0.64)
Yes	697	598	98.1	116	0.85 (0.70,1.02)	0.61 (0.56,0.66)

[a] Relatives with breast cancer investigated by the model: mother, sister/s, grandmothers (paternal and maternal), aunt/s (paternal and maternal), daughter/s. CI: confidence interval, PRS: polygenic risk score based on 313 single-nucleotide polymorphisms associated with breast cancer, IBIS: International Breast Cancer Intervention Study model (version 8b), BOADICEA: Breast and Ovarian Analysis of Disease Incidence and Carrier Estimation Algorithm model (version 5.0.0)

Supplementary Table 4: Calibration and discrimination for BOADICEA 10-year risk scores using updated Australian breast cancer incidence rates[a]

Risk model	Case-cohort, No.	Sub-cohort, No.	Expected No. of cases	Observed No. of cases	Expected /Observed ratio (robust 95%CI)	Concordance statistic (95%CI)
BOADICEA	3098	2783	362.5	408	0.89 (0.80,0.98)	0.62 (0.59,0.64)
Stratified by age						
50-64 years	1732	1549	230.5	235	0.98 (0.86,1.12)	0.65 (0.62,0.68)
65-75 years	1366	1234	132.3	173	0.76 (0.66,0.89)	0.57 (0.53,0.62)
Stratified by family history of breast cancer						
No family history	2401	2185	260.6	292	0.89 (0.79,1.00)	0.61 (0.57,0.64)
Family history	697	598	102.2	116	0.88 (0.73,1.06)	0.61 (0.56,0.66)
Stratified by quintiles of 10-year risk						
<1.7%	719	678	41.4	49	0.84 (0.64,1.12)	--
≥1.7 to 3.4%	1492	1359	152.9	173	0.88 (0.76,1.03)	--
≥3.4to 5%	553	482	89.5	99	0.90 (0.74,1.10)	--
≥5%	334	264	78.7	87	0.90 (0.73,1.12)	--

[a] Models includes age, family history, lifestyle and PRS. CI: confidence interval, PRS: polygenic risk score based on 313 single-nucleotide polymorphisms associated with breast cancer, BOADICEA: Breast and Ovarian Analysis of Disease Incidence and Carrier Estimation Algorithm model (version 5.0.0)

Supplementary Table 5: Calibration and discrimination for IBIS and BOADICEA 5-year risk scores of breast cancer[a]

Risk models	Casecohort N	Subcohort N	Expected number of cases	Observed number of cases	Expected /Observed ratio (robust 95%CI)	Concordance statistic (95%CI)
IBIS	3098	2783	223.2	213	1.05 (0.91,1.20)	0.63 (0.60,0.66)
BOADICEA	3098	2783	186.0	213	0.87 (0.76,1.00)	0.64 (0.61,0.67)
Stratified by age						
IBIS						
50-64 years	1732	1549	124.6	116	1.07 (0.89,1.30)	0.64 (0.61,0.68)
65-75 years	1366	1234	98.6	97	1.02 (0.83,1.25)	0.62 (0.57,0.67
BOADICEA						
50-64 years	1732	1549	113.0	116	0.97 (0.81,1.17)	0.66 (0.62,0.69)
65-75 years	1366	1234	73.1	97	0.75 (0.62,0.92)	0.60 (0.56,0.65)
Stratified by family history of breast cancer						
IBIS						
No family history	2401	2185	140.1	150	0.93 (0.79,1.10)	0.62 (0.59,0.66)
Family history	697	598	83.6	63	1.33 (1.03,1.71)	0.64 (0.58,0.69)
BOADICEA						
No family history	2401	2185	133.1	150	0.89 (0.75,1.04)	0.62 (0.59,0.66)
Family history	697	598	53.1	63	0.84 (0.66,1.08)	0.64 (0.59,0.70)
Stratified by quintiles of breast cancer risk						
IBIS						
<0.9%	934	868	23.9	39	0.61 (0.45,0.84)	--
≥0.9 to 1.7%	1013	934	53.7	61	0.88 (0.68,1.13)	--
≥1.7 to 2.5%	532	478	45.2	31	1.46 (1.02,2.07)	--
≥2.5%	619	503	100.5	82	1.23 (0.98,1.53)	--
BOADICEA						
<0.9%	810	756	23.4	35	0.67 (0.48,0.93)	--
≥0.9 to 1.7%	1410	1293	73.1	83	0.88 (0.71,1.09)	--
≥1.7 to 2.5%	488	419	38.8	43	0.90 (0.67,1.22)	--
≥2.5%	390	315	50.7	52	0.98 (0.74,1.28)	--

[a] Models include age, family history, lifestyle and PRS. Calibration based on number of cases with 5 or less years of follow up and discrimination based on no more than 5 years of follow up. N: number, CI: confidence interval, PRS: polygenic risk score based on 313 single-nucleotide polymorphisms associated with breast cancer, IBIS: International Breast Cancer Intervention Study model (version 8b), BOADICEA: Breast and Ovarian Analysis of Disease Incidence and Carrier Estimation Algorithm model (version 5.0.0)

Supplementary Figure 1: Melbourne Collaborative Cohort Study case cohort flowchart.[a]

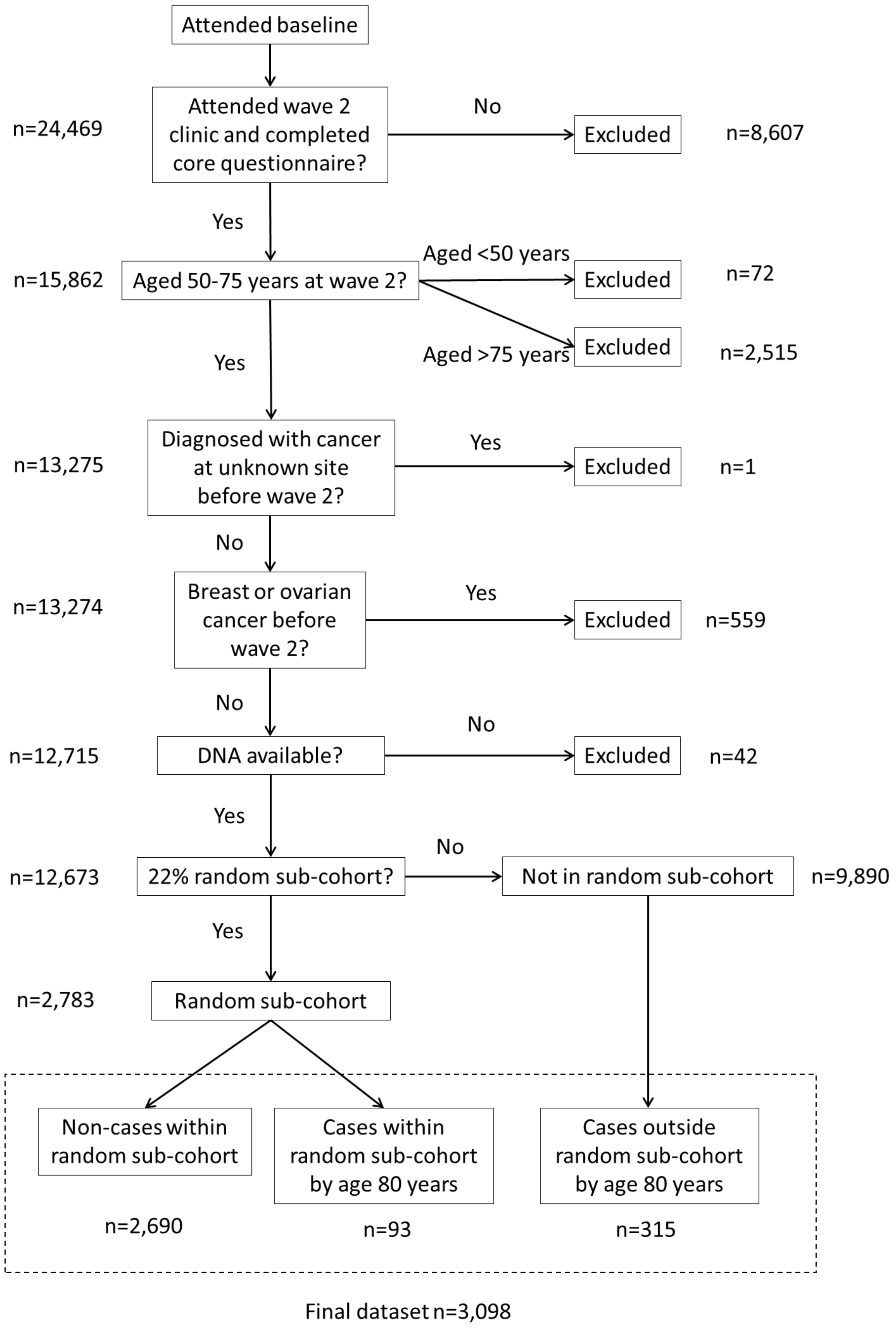

[a] N=number of participants

Supplementary Figure 2: Box plot of 10-year risk of breast cancer, comparison between BOADICEA and IBIS risk models[a]

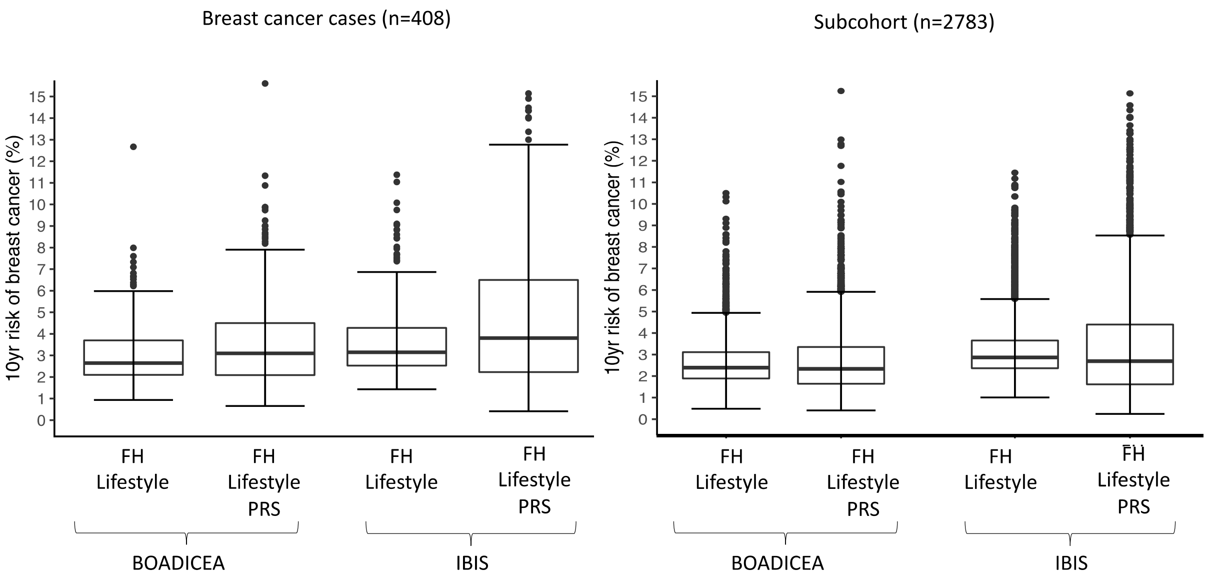

[a] Bold line within each box represents the median and borders of boxes represent the interquartile range for each risk prediction model. Sub-cohort includes 93 breast cancer cases and 2690 non-cases. n: number, FH: family history, PRS: polygenic risk score based on 313 single-nucleotide polymorphisms associated with breast cancer, IBIS: International Breast Cancer Intervention Study model (version 8b), BOADICEA: Breast and Ovarian Analysis of Disease Incidence and Carrier Estimation Algorithm model (version 5.0.0)
